# Imaging and evaluation of cervicothoracic lymphatic drainage pathways in single ventricle patients with Fontan circulation using the mDixon steady state MR angiography

**DOI:** 10.1186/s12880-025-01803-0

**Published:** 2025-07-01

**Authors:** Monika Huhndorf, Patrick Langguth, Hatim Seoudy, Olav Jansen, Anselm Uebing, Jan Hinnerk Hansen, Inga Voges

**Affiliations:** 1 Department of Radiology and Neuroradiology, University Hospital Schleswig- Holstein, Kiel, Germany; 2 Department of Internal Medicine III, Cardiology and Critical Care, University Hospital Schleswig-Holstein, Campus Kiel, Kiel, Germany; 3 Department of Congenital Heart Disease and Pediatric Cardiology, University Hospital Schleswig-Holstein, Arnold-Heller-Straße 3, Haus C, 24105 Kiel, Germany; 4 German Centre for Cardiovascular Research (DZHK), Partner Site Hamburg/Kiel/Lübeck, Kiel, Germany

**Keywords:** Fontan circulation, Single ventricle, Lymphatic vessels, 3D mDixon steady state magnetic resonanc angiography

## Abstract

**Background:**

Single ventricle (SV) Fontan patients are at risk for the development of pathological cervicothoracic lymphatic drainage pathways that are involved in the development of serious conditions such as plastic bronchitis or chylothorax. Visualization and categorization of cervicothoracic lymphatic drainage pathways might therefore help to stratify prognosis and to individualize therapy and follow-up for Fontan patients. This study aimed to show that the 3-dimensional (3D) modified Dixon (mDixon) steady state magnetic resonance (MR) angiography, commonly used to image cardiovascular anatomy, can visualize cervicothoracic lymphatic drainage pathways in high resolution in Fontan patients.

**Methods:**

3D mDixon steady state MR angiography of 88 pediatric and young adult patients with a Fontan circulation were retrospectively analysed. The pattern of cervicothoracic lymphatic pathways and image quality according to diagnostic value were assessed. Furthermore, ventricular volumes, mass and ejection fraction from cine imaging were measured.

**Results:**

Image quality was assessed as very good or good in > 90% of the cases. Six patients had a lymphatic complication of which five (83.3%) had a higher cervicothoracic lymphatic pathway type (type 3 or 4).

**Conclusions:**

3D mDixon steady state MR angiography is an established method to assess cardiovascular anatomy and function in Fontan patients. The method also allows to visualize and evaluate the cervicothoracic lymphatic anatomy with high image quality. 3D mDixon steady state MR angiography is therefore particularly useful to comprehensively assess Fontan patients, a patient group prone not only to cardiac but also lymphatic failure.

**Supplementary Information:**

The online version contains supplementary material available at 10.1186/s12880-025-01803-0.

## Background

Single ventricle (SV) patients with Fontan circulation are at risk to develop pathological changes of the cervicothoracic lymphatic system. These changes can lead to pathologies such as chylothorax or plastic bronchitis [1–4]. Due to their small calibre, visualization of lymphatic vessels on magnetic resonance imaging (MRI) is challenging. Shiina et al. and Biko et al. evaluated lymphatic drainage pathway changes in the abdomen, chest, and neck using heavily T2 weighted imaging with contrast agent for simultaneous detection of hepatocellular carcinoma or T2 weighted imaging without contrast, respectively, to visualize changes of the lymphatic system [1, 3]. Depending on the MR protocol, these imaging techniques require an additional scan, which prolongs the examination time. An MRI sequence that can visualize the lymphatic and cardiovascular system at the same time is therefore desirable, particularly for children.

Performing MR angiography often forces physicians to weigh between good contrast and high spatial resolution. Contrast enhanced MR angiography (MRA) needs fast acquisition techniques to image the target vessel at highest contrast agent concentration. Steady state angiography does not depend on fast imaging techniques and therefore enables better spatial resolution. Modified Dixon (mDixon) steady state angiography has been described as suitable for thoracic imaging in patients with congenital heart disease [5]. It allows 3-dimensional (3D) imaging with great delineation of the thoracic arterial and venous vasculature. The use of a Dixon sequence with advanced Dixon techniques also allows robust fat suppression. Robust fat suppression with other MR techniques is particularly challenging in areas with air-filled spaces like nasal and paranasal sinuses, the lung and the gastrointestinal organs [6].

Using the mDixon technique, high resolution 3D imaging with short acquisition times is possible as it uses arbitrary Time to Echo (TE)-values and therefore shortens the echo train length.

To our knowledge, the mDixon MRA has only been evaluated for visualization of venous and arterial vessels where it reached better image quality compared to balanced steady-state free precession (bSSFP) sequences and first pass contrast enhanced MR angiography (CMRA) [5, 7].

This is the first study to use the mDixon steady state MRA to evaluate the cervicothoracic lymphatic system in SV patients with Fontan circulation.

## Methods

### Patients

We included pediatric and young adult SV patients with a Fontan circulation who underwent routine cardiovascular MR imaging with a sequence protocol that included the 3D mDixon steady state MRA. The following clinical, laboratory and cardiac catheterisation data were retrospectively collected from medical records: (1) anthropometric parameters, (2) cardiac diagnoses, (3) ventricular dominance, (4) age at Fontan completion, 4) lymphatic complications, (5) NT-proBNP, serum albumin and protein levels and (6) central venous pressures in the Fontan circuit.

The study was approved by the local ethics committee (date: 20/11/2023, reference number: AZ D623/23) and performed in accordance with the Declaration of Helsinki and. A general informed consent was available for all parents or legal guardians, as appropriate.

### MR image acquisition

MRI was performed on an 3T system (Ingenia, Philips Healthcare, Best, The Netherlands). When necessary, patients were sedated with midazolam and propofol for MR examination using a standardized sedation protocol.

3D mDixon after injection of 0.1 ml/kg (= 0.1 mmol/ml) Gadolinium (Gadovist®, Bayer Vital GmbH, Germany) was used as a steady state angiography.

Standard gradient echo (GE) or steady state free precession (SSFP) cine imaging with retrospective electrocardiographic gating was performed to acquire short axis cine images to assess SV volumes, mass, ejection fraction (EF) and global function index (GFI), a new imaging marker to assess cardiac performance that can provide prognostic information for several cardiac diseases [8].

The thoracic duct in Fontan patients is typically dilated and well visualised throughout the entire chest to the point of drainage point into the venous system. In rare cases, when the thoracic duct is small and difficult to distinguish from paravertebral vessels, a static T2-weighted lymphangiography (3D Gradient and Spin Echo sequence, 3D GraSE) is performed.

### Sequence parameters

The ECG gated 3D mDixon MRA was acquired with the following parameters: Time to Repetition (TR)/1st TE/2nd TE = 4.05 ms/1.186 ms/2.37 ms; Flip angle = 10°. Due to the long period of data collection of 3.5 years, acquisition parameters show slight differences in terms of slice orientation and spatial resolution. The number of averages was set to 2 or 3 for 8% (7/88) of the patients; the remaining patients were examined with only one average. 93% (82/88) of the MRI studies were acquired in coronal view and the remainder in axial view. 92% (81/88) of the images had a reconstructed resolution of 0.75 mm³ with an acquired voxel size of 1,5 mm³. Remaining examinations were acquired with a voxel size of 1.3–1.4 mm³ and a reconstructed resolution with 0.65–0.7 mm² in plane and a spacing between slices between 0.65 and 0.75 mm. Median acquisition duration was 4:27 min (+/- 2:07 min) for 1 average, 4:44 min (+/- 1:54 min) for 2 averages and 4:40 min (+/- 0:32 min) for 3 averages. Sequence parameters are summarized in Table 1.

**Table 1:** Sequence parameters

**Parameter**	
**mDixon** TR (ms)	4.05
TE (ms)	1st image: 1.186 2nd image: 2.37
Flip Angle (°)	10
average acquisition duration (min)	4:28 (SD: 2:05)
acquired voxel size (mm)	1.3³ − 1.5³
recon voxel size (mm)	0.65³ − 0.75³
sense	RL: 2; FH:1.5
Trigger Delay	end diastole
Number of slices	75–180
Acquisition mode	cartesian
cardiac synchronization device	ECG
respiratory compensation	respiratory belt
slice orientation	Coronal (93%); axial (7%)
**Gradient echo cine imaging**	
TR (ms)	4.4
TE (ms)	2.5
Flip Angle (°)	15
Field of view (mm)	180–400
Slice thickness (mm)	5–8
Number of cardiac phases (number)	25
Temporal resolution (ms)	28.9–48.9

TR = time to repetition, TE = time to echo

Parameters for gradient echo cine imaging were as follows: slice thickness 5–8 mm (5 mm in children, 6–8 mm in adolescents and adults), 25 cardiac phases, field of view 180–400 mm, no slice gap, TR/TE 4.4/2.5 ms, flip angle 15°, temporal resolution 28.9–48.9 ms, non-breath-hold in sedated patients, breath-hold in awake patients.

### MRI analysis

#### Image quality

Two board certified radiologists with 10 years of experience rated the water-only mDixon images for image quality using a four-point consensus reading scale: 1 - very good quality without any artefacts, 2 - good quality with few artefacts that do not limit the ability to assess lymphatic drainage pathways, 3 - moderate image quality with limited ability to assess lymphatic drainage pathways, 4 - poor image quality that does not allow assessment of lymphatic drainage pathways.

#### Ventricular volumetric

SV volumetry was performed from short axis cine stacks. End-diastolic and end-systolic volumes (EDV, ESV) were measured and stroke volumes (SV), EF and end-diastolic myocardial mass (EDMM) were calculated from EDV and ESV.

Global function index (GFI) was calculated using the following formula [8]:

$$\:GFI\:=\:\left[\right(EDV-ESV)/(1/2*(EDV\hspace{0.17em}+\hspace{0.17em}ESV))+(MM/1.05\left)\right)*100]$$

#### Evaluation of lymphatic drainage pathways

**Fig. 1:**
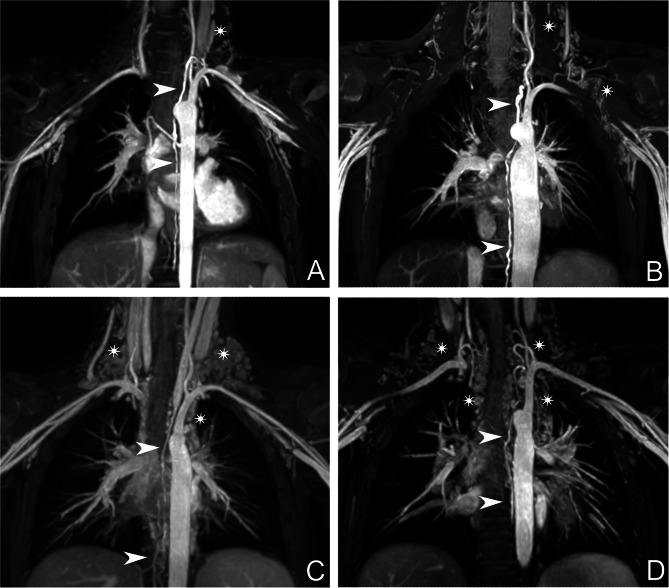
Types of cervicothoracic lymphatic abnormalities. The thoracic duct is marked with white arrows, while lymphatic abnormalities as described as type 1–4 are marked with white stars. (**A**) type 1 - little or no presumed lymphatic channels within the supraclavicular region and mediastinum, (**B**) type 2 - abnormally increased lymphatic channels within the supraclavicular region without extension into the mediastinum, (**C**) type 3 – abnormal supraclavicular lymphatics with extension into the mediastinum and (**D**) type 4 - abnormal supraclavicular lymphatics with extension into the mediastinum and in an interstitial pattern into the lung parenchyma according to Biko et al. [3]

A board certified radiologist with 10 years of experience and a board certified pediatric cardiologist with 17 years of experience in cardiac MRI evaluated steady state MRA images for the width of the thoracic duct at the supraclavicular, thoracic and upper abdominal levels, the tortuosity (straight or elongated), and the type of lymphatic abnormalities using a four-point scale according to Biko et al. with type 1 – little or no presumed lymphatic channels in the supraclavicular region and mediastinum, type 2 – abnormally increased lymphatic channels in the supraclavicular region without extension into the mediastinum, type 3 – abnormal supraclavicular lymphatics with extension to the mediastinum and type 4 - abnormal supraclavicular lymphatics with extension to the mediastinum and in an interstitial pattern into the lung parenchyma (Fig. 1) [3].

#### Statistical analysis

Statistical analysis was performed using MedCalc® (version 22.016, MedCalc Software Ltd, Belgium). The Shapiro-Wilk test was used to assess normal distribution. Normally distributed data are shown as mean and standard deviation (SD), non-normally distributed data are shown as median and 1st and 3rd quartiles (IQR) or median and range. Categorical variables were compared by chi-squared test. One-way analysis of variance was used for comparisons between categorical and continuous parameters. Adjustments for multiple testing (Bonferroni correction) were performed, and the significant p-value was reduced to 0.006.

## Results

### Patients

Patient characteristics are summarized in Table 2. Median age at the time of MRI was 8 (5.04–12.1) years. Median time between completion of the total cavopulmonary connection (TCPC) and MR examination was 66 (19–100) months.

**Table 2:** Patient characteristics

Number of patients (*n*)	88
female/male (n)	28/60
Age (y)	7.2 (3.2–23.5)
Weight (kg)* [median, IQR]	23.5 [18.0; 38.8]
MR examination in sedation (n)	70
Height (cm)* [median, IQR]	126.5 [107.5; 145.0]
Age at Fontan completion	2.0 (1.0–15.0)
*Status of Fontan completion (n)*	
TCPC	88
*Diagnosis (n)*	
HLHS	44
DORV	8
DILV	7
Tricuspid atresia	10
other	19
*Single ventricle morphology (n)*	
SRV	56
SLV	26
Both /undefined	7
*Lymphatic complications (n)*	
Chylothorax	2
Protein losing enteropathy	5
Plastic bronchitis	1
*Cardiac transplantation*	1

TCPC = total cavopulmonary connection, HLHS = hypoplastic left heart syndrome, DORV = double outlet right ventricle, DILV = double inlet left ventricle, SRV = single right ventricle, SLV = single left ventricle, y = years

*Data are expressed as median and 1st and 3rd quartile. Age is expressed as median and range

### Image quality

Image quality was classified as category 1 in 65.9% (58/88), category 2 in 27.3% (24/88), category 3 was assessed in 6.8% (6/88) and category 4 in none of the cases. Of those classified as category 1 88% were acquired with one average, 8.6% with two averages and 3.4% with three averages. Of those classified as category 2 and 3, 100% were acquired with only one average.

### Evaluation of lymphatic drainage pathways

Of the 88 patients, 31.8% (28/88) were classified as having cervicothoracic lymphatic pathway type 1, 18.2% (16/88) as having type 2, 39.8% (35/89) as having type 3 and 9.1% (8/88) as having type 4.

Width of lymphatic duct on the three assessed levels differed hardly with a median of 2.4 (2.1–2.7) mm on the supraclavicular level, 2.4 (2.2–2.65) mm on the thoracic level and 2.3 (2-2.6) mm on the abdominal level. Course of lymphatic duct was straight in 83.0% (73/88) and elongated in 17.0% (15/88).

### Clinical and MR findings and lymphatic status

Six patients had a lymphatic complication with pleural effusion (5/88), protein losing enteropathy (3/88), chylothorax (2/88) and/or plastic bronchitis (1/88). Five out of these six patients had higher lymphatic categories (type 3 or 4, 83.3%), whereas only one patient had a lower category (type 1 or 2, 16.7%).

There was no association between single ventricle morphology (*p* = 0.06), age at TCPC (*p* = 0.32), protein levels (*p* = 0.21), NT-proBNP results (*p* = 0.73) or central venous pressures (*p* = 0.64) with the cervicothoracic lymphatic pathway category.

No relationships between lymphatic status and SV volumes, EDMM, EF or GFI were detected (*p* > 0.05 for all).

## Discussion

MR lymphatic imaging has recently been added to the diagnostic armamentarium used in the follow-up of Fontan patients typically by adding T2-weighted non-contrast MRI imaging to the sequence protocol. We could show that the mDixon steady state MRA allows detailed delineation and evaluation of the cervicothoracic lymphatic system in patients with the Fontan circulation. Since acquisition of mDixon images is part of the routine MR examination, no extension of the MR protocol is needed to detect lymphatic abnormalities using this technique.

Patients with lymphatic complications had a higher lymphatic category, showing that the mDixon sequence can detect patients at risk for lymphatic diseases.

In the described protocol, the mDixon images were acquired immediately after contrast agent injection for optimal image quality for precise evaluation of the cardiovascular anatomy [9, 10]. As expected, image quality was graded higher when a larger number of averages was used. But even with only one average a very good quality without any artefacts was achieved in 63% of the scans.

Other angiographic MR techniques, especially the 3D-balanced steady state free precession (3D-bSSFP) sequence, have been used to assess the cervicothoracic lymphatic pathway in SV patients. Studies could show that the 3D-bSSFP in combination with T2-weighted (T2w) lymphangiography can improve lymphatic imaging. However, tracking of the complete thoracic duct was only possible in 54% of the included patients [11]. Castellanos et al. could visualize the thoracic duct in two-thirds of their patients with a Fontan circulation on 3D-bSSFP images [12]. With the use of the 3D mDixon sequence in our study, the cervicothoracic ductal pathway could be well visualized in 93% of patients.

With the increasing knowledge of the pathophysiology of lymphatic diseases in Fontan patients [2], MR assessment of the lymphatic system is of growing clinical importance in this patient group [3, 13]. Besides describing lymphatic patterns before the Glenn operation or Fontan completion and relating them to clinical outcome and events [3, 14, 15], recent studies have shown that quantification of the lymphatic burden with T2w lymphangiography is possible and that a higher burden seems to be associated with an adverse outcome [16]. Furthermore, MR-based techniques can be used to guide lymphatic interventions [17–19]. Using the mDixon technique, we found that patients with lymphatic complications had a higher lymphatic scale according to classification suggested by Biko et al. (Lit). Our study therefore supports the notion, that this classification system is useful to detect Fontan patients that are at a particular risk for lymphatic failure and disease.

Finally, given the high image quality and the robustness of the mDixon sequence, the technique lends itself to routine and serial evaluation of the lymphatic system. The latter is needed to gain further insights into the pathophysiology, prognostic importance and treatment options of lymphatic diseases of Fontan patients.

## Limitations

This study has several limitations including its retrospective design. Firstly, we did not systematically combine the mDixon method with T2-weighted lymphangiography, the current standard method for assessing lymphatic vessels, in most patients, as the image quality of the mDixon steady-state MRA was good or very good in 93% of patients and classification of lymphatic abnormalities was possible in all patients. Nevertheless, in few patients the course of the thoracic duct and the lymphatic pattern was confirmed by T2-weighted lymphangiography (3D GraSE, see Additional file 1). However, prolonging an MR examination by adding a T2-weighted lymphangiography particularly in mostly sedated children did not seem indicated.

Secondly, abdominal lymphatic abnormalities were not assessed.

## Conclusions

The 3D mDixon steady state MRA allows visualization and classification of the cervicothoracic lymphatic pathways in patients with the Fontan circulation with high image quality. The technique can therefore be considered ideal for serial follow-up of Fontan patients in whom not only the arterial and venous vasculature but also the lymphatic systems is abnormal and cause for disabling and life-threatening disease.

## Electronic supplementary material

Below is the link to the electronic supplementary material.

Supplementary Material 1

## Data Availability

The data that support the findings of this study are not openly available due to reasons of sensitivity and are available from the corresponding author upon reasonable request.

## Abbreviations

bSSFP: Balanced steady-state free precession
EDMM: End-diastolic myocardial mass
EDV: End-diastolic volume
EF: Ejection fraction
ESV: End-systolic volume
GE: Gradient echo
GFI: Global function index
IQR: Interquartile range
MRA: Magnetic resonance angiography
mDixon: Modified Dixon
MRI: Magnetic resonance imaging
SV: Single ventricle
TCPC: Total cavopulmonary connection
TE: Echo time
TR: Repetition time
3D: Three-dimensional
