# Supplementary figures and images for: Imaging and evaluation of cervicothoracic lymphatic drainage pathways in single ventricle patients with Fontan circulation using the mDixon steady state MR angiography
Source: BMC Med Imaging. 2025 Jul 1;25:229. doi: 10.1186/s12880-025-01803-0 (PMC12211272; doi:10.1186/s12880-025-01803-0)

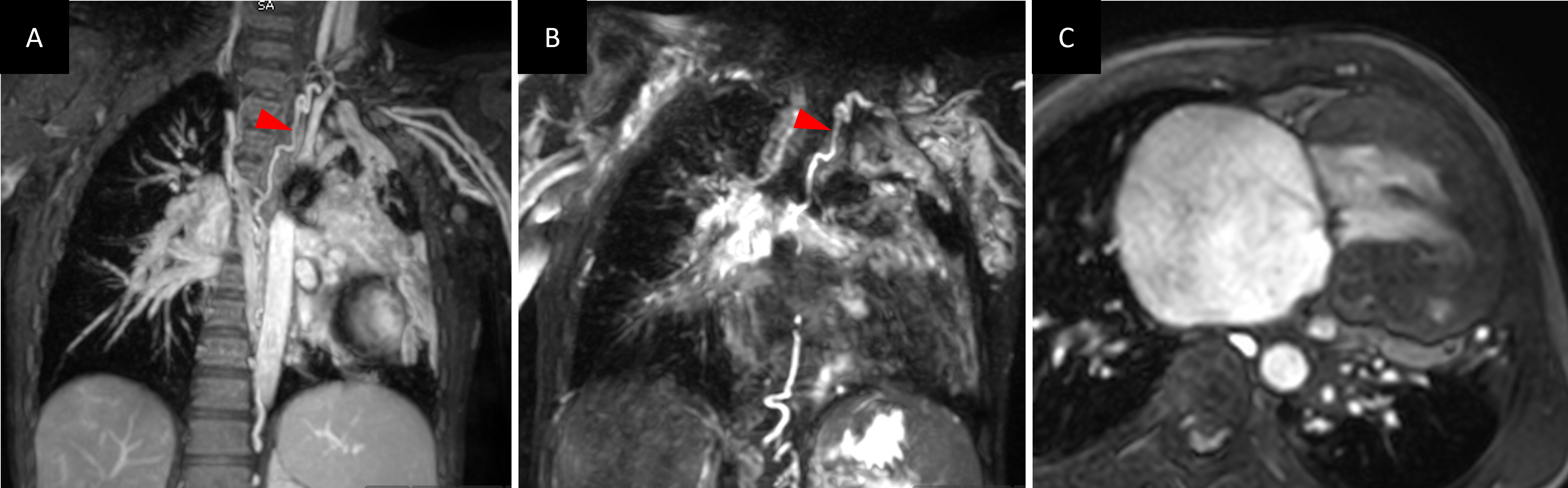

Supplement: Supplementary file 1 — Supplementary Material 1 [file 12880_2025_1803_MOESM1_ESM.tif]
